# Towards optimising children’s capability and tackling relative child poverty in high-income countries: the cases of Japan, Sweden and the UK since 2000

**DOI:** 10.1080/16549716.2022.2084230

**Published:** 2022-07-18

**Authors:** Hajime Takeuchi, Sung-Hee Lee, Anneli Ivarsson

**Affiliations:** a School of Social Welfare, Bukkyo University, Kyoto, Japan; b Department of Epidemiology and Global Health, Umeå University, Umeå, Sweden; c Department of Social Sciences, University of Derby, Derby, UK

**Keywords:** Children’s capability, high-income countries, relative child poverty

## Abstract

**Background:**

We question why child poverty still prevails even in high-income countries, such as Japan, Sweden and the United Kingdom. We address the intersection between social relations and individual experiences that should be considered when optimising children’s capability.

**Objectives:**

The study is therefore aimed at exploring compensatory societal actions taken to optimise children’s capability among these affluent countries. In order to do so, we operationalised children’s capability by including key societal domains along with statistical indicators and variables from relevant sources.

**Methods:**

A secondary quantitative method was adopted by drawing upon data sources from 2000 up to almost 2020 from the Organisation for Economic Co-operation and Development, the World Bank and the United Nations Children’s Fund, with these being complemented by governmental data. Given a lack of currently available and comparable data for those three countries, four key societal domains were explored in an absolute descriptive analysis.

**Results:**

It is obvious that child poverty prevailed over the focal 20 years in these three high-income countries. Also, the exploratory data analysis revealed a lack of sufficient supporting social services in each societal domain. This demonstrates that optimising children’s capability should not just be about subsidising economic resources, but also supporting all initiatives aimed at addressing the lack of interactions between each domain of children’s capability.

**Conclusions:**

The study shows how essential it is to consider societal compensatory measures along with supporting the financial circumstances. We therefore argue that optimising children’s capability should not only be about subsidising economic resources, but also ensuring adequate social resources and relations.

## Background

Whilst Sen argues that someone’s capability reflects ‘the alternative combinations of functionings the person can achieve’, more importantly it should be concerned with their actual ability to achieve various valuable functionings as a part of their life [1,2]. Children’s capability can only be optimised through a holistic approach that allows them to make valued choices for their own well-being as well as to achieve their own wishes and goals within their society [3–5]. In this study, we use the meaning of ‘optimising’ not as a fixed matter, but rather a concept that depends on the child and their surroundings. In other words, optimising children’s capability is built upon the relationship between society and the understanding of children’s will i.e. what they want to ‘be’, ‘do’, or ‘become’. In order for them to do so, children should have ‘choice’ and ‘freedom’, with equal access and opportunities to what they value, rather than being limited by the resources they are given [6–8]. Yet, poverty often leads to their being deprived of certain capabilities, which can vary from basic physical needs for nourishment and shelter to more complex social dimensions [9].

Child poverty is usually measured in either absolute or relative terms. While absolute poverty assessment tends to focus on essential basic needs, such as enough food, decent housing, water and so on, relative poverty emphasises more the deficit in social arrangements or not being able to fully take part in one’s society [10]. The threshold for relative poverty has been defined by the Organisation for Economic Co-operation and Development (OECD) as households receiving 50% less than the median household income in a specific country [11]. Thus, they do have some money, but still they may not be able to fully take part in the life of the community nor appear in public without feeling a sense of shame [12].

When interrogating children’s capability and acknowledging the relative deficit of social resources, a lack of wider social relations that individual children could possibly experience in their lifetime due to their deprivation is considered. Lister [13,14] defined poverty as not only being about material disadvantages, but also, social ones, which can be experienced due to the lack of interactions with the wider society. Whilst the relationship aspect of an individual’s well-being is stressed, this should not be only about interpersonal matters, but also relations with the community and wider social structures. Gupta added that children, especially those with ‘inferior’ parents, tend to be categorised as disadvantaged, however, the process of ‘othering’ and their lack of social relations has been neglected in academic discussion of child poverty [9]. We, therefore, argue that the intersection between limited social relations and individual shame should be considered when exploring the concept of children’s capability.

As aforementioned, we selected three high-income countries for our analyses, i.e. Japan, Sweden and the United Kingdom (UK), for the following reasons. First, relative child poverty in these economically leading countries has prevailed over the last few decades [15]. For example, in Japan, nearly half of children in single-parent families experience poverty [16]. In the UK, 4.2 million children were living in poverty in 2018–2019, this being 30% of all children or nine out of a classroom of 30 [17]. Also, children in the UK living in lone-parent families as well as those from black and ethnic minority groups are more likely to live in poverty [18]. In Sweden, the European Union definition of relative child poverty is mostly used, i.e. defined as a household with income lower than 60% of the median, which in 2014 was 15%, while it was 18% in Japan and 20% in the UK [19]. With no doubt, these countries are all wealthy compared to many less developed countries globally, but child poverty still prevails.

This continuing child poverty demonstrates a failure in these countries with respect to effective policy responses to tackle the problem. The Japanese government declared that it would eradicate child poverty through the introduction of a universal childcare allowance in 2009 [20]. However, this did not bring any significant change for families with two or more children and/or single-mother households. In fact, the poverty rate of the latter has increased by 50% over the last 25 years [21]. In 2010, the UK government, with cross-party support, passed the Child Poverty Act, aimed at eradicating child poverty by 2020. However, this political effort seems to have been nullified when subsequent governments introduced austerity measures, such as freezing child benefit [22]. Relative child poverty in Sweden has been tackled by an active family-oriented economic policy that includes financial transfers to families with children. However, whilst since 2000 the annual median income has been increasing for all income groups, including families with children, these redistributing systems have not been sufficiently effective. Rather, economic inequity has increased in Swedish society at large, and is manifested also by increased relative child poverty [23].

Secondly, by focusing on developed and affluent countries, we aim to discuss children’s capability as an entitlement of social justice. These three countries have been categorised as having different types of welfare-state regimes [24]. Japan has ‘key elements of both the liberal-residual and the conservative-corporatist model’, while the UK relies heavily on private welfare provision as a liberal welfare state [25]. In contrast, Sweden is a social-democratic welfare state, with universal citizen-related benefits and rights. This dissimilar typology of welfare-state regimes is salient when examining the spending on welfare provision as well as the coverage and generosity of the distributed benefit [26]. This leads us to our research questions: why does child poverty still prevail regardless of the welfare provision and what can be done to reduce it? What compensatory measures can be put in place to optimise children’s capability among these wealthy countries? We believe that these high-income countries have better functioning democracies and institutions, with a higher value being placed on social justice, when compared to many other states, where often almost no infrastructure and only minimal state support is available [5]. In sum, this study is aimed at assessing children’s life circumstances in Japan, Sweden and the UK, whilst exploring compensatory societal actions taken to optimise children’s capability in these affluent countries. In order to do so, we have developed our analytical framework, which operationalises children’s capability by including key societal domains along with statistical indicators and variables. We have then employed a secondary quantitative method, which involved drawing upon data sources since 2000 from relevant international organisations, with these being complemented by governmental data.

## Research methodology

Our starting point is the human right of each child from birth onwards to have life conditions that optimise their capability, with respect to all aspects of their development: cognitive, physical, social and emotional. For most children, this will pave the way for good educational achievement and a prosperous life, but irrespective of success in this regard or not, every child should be afforded with dignity and respect.

In order to operationalise this, we refer to the studies by Biggeri and Mehrotra and Schweiger and Graf, and have thus included four societal domains: family, education, social security and social economy [5,27]. The domain ‘*family*’ is a place where children’s life and health should be guaranteed with love and care, whilst also being protected from any violence. The domain ‘*education*’ encompasses social relations in which children can participate in and influence, while also receiving age-adapted objective information. The domain ‘*social security*’ should provide children with freedom from economic and non-economic exploitation, which also involves a healthy, safe and pleasant environment. Lastly, we consider the domain of ‘*social economy’*, which should provide children with leisure activities regardless of their economic situation and ensure that they are respected and treated with dignity.

When determining relevant indicators for each domain, we had to address several methodological challenges. First, we decided to use *integrated indicators* for each dimension, instead of dividing them into the macro and micro levels. This is because children’s capability is influenced by a combination of institutional-based resources provided at the macro-level and the actual conditions, where each social arrangement is available for them at the micro-level. In fact, this methodological challenge was a concern of Sen too, because providing a fixed list of capabilities could be ‘to deny the possibility of fruitful public participation on what should be included and why’. In accord with his disinclination to use a fixed list of indicators to measure the capability [1], the integrated indicator approach enabled us to consider aspects that might influence children’s capability either directly or indirectly at both levels. Secondly, we focused on *preconditional indicators* in relation to children’s capability, rather than outcomes. Such indicators should reflect the financial situation of the family, including societal measures aimed at supplementing its economic resources. Importantly, compensatory societal measures that could promote children’s capability also needed to be considered. By focusing on preconditional indicators, we aim to produce policy implications at the end of this paper.

Thirdly, when faced with the challenge of comparing three countries, i.e. Japan, UK and Sweden, we decided to do use secondary quantitative data for the time period from the year 2000 up until the present. While the motives for the selection of countries have been given above, the reason for starting in the year 2000 was that, since then, the world, through the United Nations, has increased its ambition of giving every child a good life by ending poverty in all its forms everywhere: first, through the Millennium Development Goals and more recently, through Agenda 2030 and its Sustainable Development Goals [28]. As aforementioned, the data sources are from well-established international organisations, including the OECD, the World Bank and the United Nations Children’s Fund (UNICEF), as well as being complemented by governmental data. However, the selection of indicators was limited by data availability, both with respect to indicators as such and the time period covered. Importantly, most indicators primarily concern adults, but are also expected to influence the lives of the children living with them, thus, influencing the latter’s capability. Also, when possible, we have reported indicators separately for men and women, as women are usually closer to the children and hence, their life circumstances more likely to influence their capabilities.

**Figure 1.**
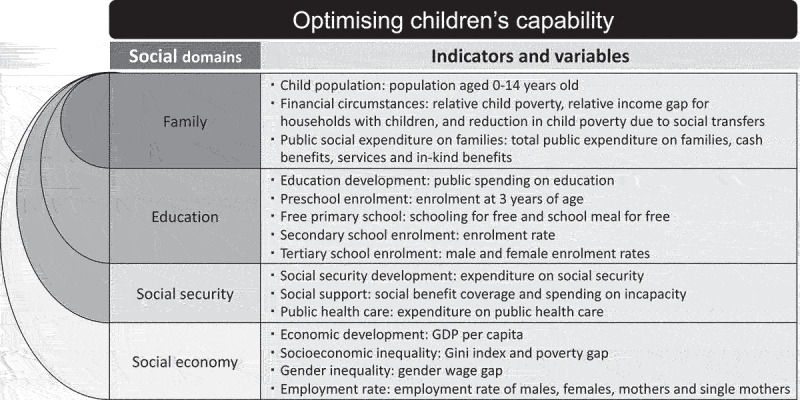
The analytical framework. The analytical framework is with societal domains of importance for children’s capability and with each domain being operationalised by certain indicators and variables

Figure 1 below sets out the analytical framework for this study, with societal domains of importance for children’s capability and with each domain being operationalised by certain indicators and variables. Due to the lack of comparable available data among the selected three countries, we had to restrict our indicators and the data analysis to only comparing absolute indicator values as an exploratory inquiry, rather than being able to present any correlations or the effects of the indicators and variables.

## Results

### The family domain

Notably, the proportion of children (0–14 years) of the total population differed between the countries, with Japan being at 15%, compared to Sweden at 18% and the UK at 19% (2017) [See Table 1]. The family financial circumstances in each country are illustrated according to the level of relative child poverty, relative income gap for households with children, and reduction in child poverty due to social transfers. In Japan and the UK, the child poverty level has largely remained unchanged over the study period from 2000 until 2018, at 14% and 13%, respectively. Comparing the three countries, Sweden had the lowest level in 2018, at 9.3%; however, this does mean that it has doubled since 2000. The relative income gap for households with children reveals how much the poorest children were left behind, and here, Japan was worst off [60%], followed by Sweden [46%] and then, the UK [40%]. All countries had in place social transfers to families with the purpose of reducing child poverty; however, less so in Japan, with only 18% compared to Sweden and the UK, with 55% and 54%, respectively.

**Table 1.** The family domain illustrated by indicators and data from 2000 onwards.

Indicators	Variables	Countries						Sources
		Japan		Sweden		UK		
Child population^1^	Population aged 0–14 y (% of total population)	13	[2017]	18	[2017]	18	[2017]	World Bank Open Data
		15	[2000]	18	[2000]	19	[2000]	
Financial circumstances	Relative child poverty ^1^ (%)	14	[2018]	9.3	[2018]	13	[2018]	Various^2^
		14	[2000]	4.2	[2000]	14	[2000]	
	Relative income gap for households with children ^3^ (%)	60	[2013]	46	[2013]	40	[2013]	Unicef
	Reduction in child poverty due to social transfers (%)	18	[2014]	55	[2014]	54	[2014]	Unicef
Public social expenditures on families	Total (% of GDP ^4^)	1.6	[2017]	3.4	[2017]	3.2	[2017]	OECD Stat.
		0.6	[2000]	2.8	[2000]	2.4	[2000]	
	Cash benefits (% of GDP)	0.7	[2017]	1.2	[2017]	2.1	[2017]	OECD Stat.
		0.2	[2000]	1.4	[2000]	1.6	[2000]	
	Services and in-kind benefits (% of GDP)	0.9	[2017]	2.2	[2017]	1.1	[2017]	OECD Stat.
		0.4	[2000]	1.4	[2000]	0.8	[2000]	

1. According to the OECD, the definition is ‘the ratio of the number of people whose income falls below half the median household income’ [11].

2. Several sources were used: Japan, Japanese Government for both 2000 and 2018; Sweden, Unicef for 2000 and OECD for 2018; and the UK, OECD for both 2000 and 2018.

3. Difference in income between households at the 10^th^ and 50^th^ percentile.

4. Gross Domestic Product (GDP) per capita is a country’s average economic annual output per inhabitant.

In all three countries, public social expenditure on families increased over the focal time period, but for Japan, this remained at a considerably lower level, with 1.6% of Gross Domestic Product (GDP) being allocated compared to 3.4% in Sweden and 3.2% in the UK. The mode of support differed between the countries, with Japan dividing it equally between cash benefits and services, while Sweden prioritised services and in-kind benefits and the UK prioritised cash benefits.

### The education domain

Public spending on education in the three countries over the period from 2000 to 2017 was within the range of 3.2% to 7.6% of GDP [*See* Table 2]. Japan remained in the lower range during the whole period, while the UK increased its investments from 4.0% to 5.4% and Sweden from 6.7% to 7.6% of GDP. In Japan and Sweden, compulsory school is nine years, while it is 13 years in the UK. Primary and secondary school is free of charge in all three countries. In Sweden, the schools are obliged to provide a prepared meal for lunch free of charge for all children, which is not the case in the other two countries.

**Table 2.** The education domain illustrated by indicators and data from 2000 onwards^1.^

Indicators	Variables	Countries						Sources
		Japan		Sweden		UK		
Education development	Government spending on education	3.2	[2017]	7.6	[2017]	5.4	[2017]	World Bank Open Data.
	(% of GDP ^2^)	3.5	[2000]	6.7	[2000]	4	[2000]	
Preschool enrolment	Enrolment at 3 years of age	83	[2018]	92	[2018]	100	[2018]	Governments
		81	[2013]	93	[2013]	97	[2013]	
Primary school	School for free	Yes		Yes		Yes		Governments
	School meal for free^3^	No		No		No		
Secondary school enrolment	Enrolment (%)	97	[2015]	100	[2015]	98	[2015]	Sweden/UK:
								World Bank Open Data.
		96	[2000]	96	[2000]	95	[2000]	Japan: Government
Tertiary school enrolment	Male enrolment (%)	52	[2018]	56	[2018]	52	[2018]	Sweden/UK:
								World Bank Open Data.
		40	[2000]	55	[2000]	54	[2000]	Japan: Government
	Female enrolment (%)	58	[2018]	90	[2018]	71	[2018]	Sweden/UK:
								World Bank Open Data.
		32	[2000]	80	[2000]	63	[2000]	Japan: Government

1. Compulsory education: Japan, 9 years, from age 6 to 15; Sweden, 9 years, from age 7 to 16; UK, 13 years, from age 5 to 18.

2. Gross Domestic Product (GDP) per capita is a country’s average economic annual output per inhabitant.

3. Only Sweden has school meals for free for every child. In Japan and the UK, it is only free for children from low-income families.

Enrolment in preschool at three years of age was high in all three countries, but in Japan, nearly one fifth of the children remained at home. Secondary school enrolment was high in all three countries, ranging between 97% and 100% in 2015. Moreover, they all experienced increased female enrolment in tertiary school, most prominent in Japan, where in 2000 this was only 32% and by 2018 had reached 58%. In that year, female enrolment in Sweden registered 90% and 71% in the UK. Male enrolment in tertiary school was at a lower level in all three countries, with Japan and UK on 52% and Sweden 56%.

### The social security domain

Spending on social security was highest, at 26% of GDP in Sweden in 2017, which had experienced no increase since 2000, while it increased over time in both Japan and UK to reach 22% and 20%, respectively [*See* Table 3]. Spending on social security includes a wide range of social support, such as pensions, elderly care, family support, and health care [29]. Notably, over time, all three countries had a change in population structure towards more elderly (aged 65 years and above). This was most pronounced in Japan, with an increase from 17% in 2000 to 27% in 2017, compared to 17% to 20% in Sweden and 16% to 18% in the UK.

**Table 3.** The social security domain illustrated by indicators and data from 2000 onwards^1^.

Indicators	Variables	Countries						Sources
		Japan		Sweden		UK		
Social security development	Expenditure on social security ^2^ (% of GDP ^3^)	22	[2017]	26	[2017]	20	[2017]	OECD Stat.
		15	[2000]	26	[2000]	17	[2000]	
Social support	Social benefit coverage^4^ in persons below 65 years (%)	0.8	[2016]	2.8	[2016]	1.9	[2016]	OECD Stat.& World Bank
		0.5	[2007]	2.8	[2007]	4.1	[2007]	
	Spending on incapacity ^5^ (% of GDP)	1.8	[2017]	3.8	[2017]	1.9	[2017]	OECD Stat.
		0.7	[2000]	4.8	[2000]	2.2	[2000]	
Public health care	Expenditure on public health care (% of GDP)	9.2	[2018]	9.3	[2018]	7.8	[2018]	OECD Stat.
		5.8	[2000]	6.3	[2000]	5.6	[2000]	

1. In 2000 and 2017, the proportions of the population aged 65 and above were 17% and 27% in Japan, 17% and 20% in Sweden, and 16% and 18% in the UK, respectively.

2. Total social expenditure for all ages, including pension, elderly care, family support, social support, health care, etc.

3. Gross Domestic Product (GDP) per capita is a country’s average economic annual output per inhabitant.

4. Social benefit is the livelihood protection for those below 65 years. This is based on OECD Stat and World Bank data, which has been recalculated to be comparable between the countries.

5. Incapacity refers to sickness, disability and occupational injury.

Social benefits as livelihood protection were in place in all three countries; however, the system and coverage differed considerably. The social benefit coverage in 2016 for those below 65 years was only 0.8% in Japan, while it was 2.8% in Sweden. Notably, in that year it was only 1.9% in the UK, having decreased from 4.1% in 2007. Spending on incapacity, i.e., sickness, disability and occupational injury, differed considerably amongst the three countries over the period in question. In 2017, Japan and the UK spent 1.8% and 1.9% of GDP, respectively, while Sweden spent double that at 3.8%.

Public health care expenditure increased in all three countries between 2000 and 2018, reaching 9.2%, 9.3% and 7.8% of GDP in Japan, Sweden and the UK, respectively. All these countries have an ageing population, most so for Japan, which implies that quite a large part of the health expenditure was used for that age group [30]. In Japan, part of the health care cost was out-of-pocket payment, in contrast to Sweden and UK, where it was almost free of charge.

## The social economy domain

All three countries experienced positive economic development over the period from 2000 to 2018 [*See* Table 4] however, this was less so in Japan compared to Sweden and the UK. Sweden was socioeconomically more equal compared to the other countries, as reflected in both the Gini Index at 0.28 and the poverty gap at 0.23, illustrating the wealth gap and severity of poverty, respectively. Japan and the UK had a Gini Index in 2018 of 0.33 and 0.37, respectively, which remained largely unchanged over the studied period. In both these countries, the poverty gap is higher than in Sweden, being 0.36 in Japan and 0.37 in the UK. The poverty gap in Sweden has slightly decreased over time, while it has remained unchanged in Japan and increased in the UK.

**Table 4.** The social economy domain illustrated by indicators and data from 2000 onwards.

Indicators	Variables	Countries						Sources
		Japan		Sweden		UK		
Economic development	GDP per capita (USD) ^1^	41,336	[2018]	53,747	[2018]	46,956	[2018]	World Bank Open Data
		26,839	[2000]	29,629	[2000]	26,413	[2000]	
Socioeconomic inequality	Gini index ^2^	0.33	[2018]	0.28	[2018]	0.37	[2018]	OECD Stat.
		0.34	[2000]	0.24	[2000]	0.35	[2000]	
	Poverty gap ^3^	0.36	[2018]	0.23	[2018]	0.37	[2018]	OECD Stat.
		0.36	[2000]	0.26	[2000]	0.28	[2000]	
Gender inequality	Gender wage gap (%) ^4^	24	[2019]	7.6	[2019]	16	[2019]	OECD Stat.
		34	[2000]	12	[2000]	26	[2000]	
Employment ^5^	Male (%)	84	[2018]	79	[2018]	80	[2018]	OECD Stat.
		81	[2000]	76	[2000]	79	[2000]	
	Female (%)	70	[2018]	76	[2018]	71	[2018]	OECD Stat.
		57	[2000]	72	[2000]	66	[2000]	
	Mothers ^6^ (%)	71	[2018]	86	[2018]	73	[2018]	OECD Stat.
		57	[2005]	81	[2005]	65	[2005]	
	Single mothers (%)	82	[2016]	80	[2016]	64	[2016]	Sweden/UK: OECD Stat. Japan: Government
		81	[2011]	65	[2011]	52	[2011]	

1. Gross Domestic Product (GDP) per capita is a country’s average economic annual output per inhabitant expressed in US Dollars.

2. Gini index: the income distribution of a nation’s residents, ranging from 0–1, with increasing inequality with higher values.

3. Poverty gap: the ratio by which the mean income of the poor falls below the poverty line, the latter being 50% of the median income in the population.

4. Gender wage gap (%): defined as the difference between the median earnings of men and women working full time.

5. Employment population ratio (%) among those aged 15–64 years.

6. Mother’s employment concerns women with children below 18 years of age.

Gender inequality was represented by the wage gap, i.e. the difference in the median earnings of men and women working full time. Over time, the gender wage gap has decreased in all three countries, however, it has remained considerably higher in Japan [24%], compared to the UK [16%] and Sweden [7.6%]. Men in all three countries were employed more often than women. However, over time women have been increasingly taking part in the labour market, with the largest increase in Japan, from 57% to 70%. In Sweden, women are approaching the same level of employment as men, standing at 76% and 79%, respectively. Among single mothers, employment in Japan was high during the whole period studied [about 80%], while it increased over time in both Sweden and the UK to 80% and 64%, respectively.

## Discussion

In this study, we have pursued the perspective of children’s capability when questioning why child poverty is still rife even in high-income countries, such as Japan, Sweden and the UK. We have examined the level of relative child poverty and any changes over time, whilst also exploring compensatory societal actions taken to optimise children’s capability. Four key societal domains of children’s capability were explored: family, education, social security and social economy. We argue that optimising children’s capability should not only be about subsidising economic resources, but also, ensuring adequate social resources and relations. This means that, whilst the family should be a place where children’s lives and physical health should be guaranteed, with love and care, they should also have access to adequate resources and social relations. Through a holistic approach to children’s capability, we raise three discussion points with regard to tackling relative child poverty and optimising their capability, especially in high-income countries.

First, findings from the family domain have clearly shown that, whilst relative child poverty needs to be tackled by giving financial support to families, it is important to go beyond this and implement compensatory societal actions. Notably, the relative child poverty in Japan was unchanged during the study period [14%], despite the percentage of GDP spent on public social expenditure having more than doubled. Similarly, in Sweden and the UK, in spite of the percentage of GDP spent on public social expenditures having increased over the focal period, relative child poverty doubled in Sweden and remained unchanged in the UK. It is also intriguing to see that, whilst the percentage of GDP for cash benefits increased over time in both Japan and the UK, the relative child poverty remained largely unchanged. By contrast, in Sweden, the cash benefits remained unchanged, whereas the services and in-kind benefits increased over this period. Thus, it is important to explore how children’s capability can be best promoted considering the share of support given by cash benefits, services and in-kind benefits.

Second, the findings from the domains of education and social security revealed the importance of the intersection between persistent child poverty and children’s lack of social relations and social resources. Without doubt, social security should guarantee children’s economic independence, thus preventing economic and non-economic exploitation, as well as for their being able to have a healthy, safe and pleasant environment. In Japan and the UK, the spending on social security as a percentage of GDP increased over the study period, while it remained unchanged in Sweden. However, the lattermost country still had the highest level of spending on both total social security and social benefit coverage for persons below 65 years.

Third, the social economy domain findings tell us that, while there was positive economic development over the focal years in all three countries, inequality increased in Sweden, as reflected by the Gini Index and the poverty gap widened in the UK. A clear gender wage gap with female disadvantage prevailed in all three countries, however, this was considerably wider in Japan and the UK than in Sweden. Employment has been going up over time for females in all three countries, but is still lower than for male workers. Single mothers in Japan and Sweden work considerably more often than in the UK. Notably, in Japan, the public social expenditure on families is considerably lower than in the other countries, which has most likely contributed to the high proportion of single mothers that were employed. Considering the large gender wage gap in Japan, with women being disadvantaged, these families are more exposed to being financially vulnerable and this could limit their children’s capability.

In taking the four societal domains as a holistic approach to children’s capability, we were able to identify preconditional variables that could influence children’s capability. The available data provided us with a platform for examining how, given the current circumstances, children’s capability can be optimised. Nonetheless, we have to acknowledge the lack of appropriate data to examine the phenomenon in full. In fact, the limitations of data are not entirely prohibitive, as ‘having some knowledge of a few functionings is always better than having none’ [5].

## Conclusion

The lack of comparable data among the selected countries remains a limitation of this study. We, however, argue that in policy debates about optimising children’s capability, the intersection between individual financial circumstances and compensatory societal support should be prioritised. Thus, societal compensatory measures are needed along with financial support being provided to economically deprived families with children.

The different types of welfare regimes among the focal countries might have distinctive policy legacy, and therefore, different policy contexts and priorities. We argue that regardless of welfare regime it matters greatly to make our societies more equal through redistributing economic resources as well as advocating the equal right for the individual child to be recognised as being independent and having dignity. For example, the connectivity between individual financial circumstances and limited social relations should be prioritised in policy debates, when exploring the concept of children’s capability. This should also be aligned with the ambitions of Agenda 2030 and its Sustainable Development Goals [UN 2015] in leaving no child behind. In sum, the focus should be on eradicating child poverty by redistributing economic resources, and by strengthening key compensatory measures to optimise individual children’s capability.

## Paper context

We assessed relative child poverty and other life circumstances of children in the three high-income countries of Japan, Sweden and the UK using publicly available data. The findings have illustrated how it is essential to consider possible compensatory societal measures along with the supporting financial circumstances. We have concluded that the focus needs to be on eradicating child poverty by redistributing economic resources, while also strengthening key compensatory measures aimed at optimising individual children’s capability.
